# A double dilemma: treatment of stage IV fetal twin-twin transfusion syndrome in the setting of maternal recurrent venous thromoembolism: a case report

**DOI:** 10.1186/s12884-019-2551-9

**Published:** 2019-10-24

**Authors:** Claire M. McCarthy, Alya al-Madhani, Suzanne Smyth, Nóirín E. Russell, Ruwan Wimalasundera, Keelin O’Donoghue

**Affiliations:** 1 Department of Obstetrics and Gynaecology, Cork University Maternity Hospital, Wilton, Cork, Ireland; 2 Department of Obstetrics and Gynaecology, Royal Hospital, Muscat, Oman; 3 Fetal Medicine Unit, University College London Hospital, 1st Floor EGA Wing, 235 Euston Road, London, NW1 2BU UK; 4 Irish Centre for Maternal and Child Health Research (INFANT), University College Cork, Cork, Ireland

**Keywords:** Fetoscopy, Fetoscopic laser photocoagulation, Multi-disciplinary care, Twin twin transfusion syndrome, Venous Thromboemobilic disease

## Abstract

**Background:**

Fetal conditions can pose significant challenges in the management of pregnancies complicated by pre-existing maternal medical conditions.

**Case presentation:**

We report a case of a 34-year-old woman with Stage IV Twin Twin Transfusion syndrome in the presence of maternal recurrent complex venous thromboembolic disease. Following a previous pregnancy loss, complicated by a third episode of thromboembolic disease, an inferior vena cava filter was placed. One month later, a pregnancy was confirmed and subsequently identified as a monochorionic twin pregnancy. Twin-Twin Transfusion syndrome was identified at 18 weeks’ gestation and progressed rapidly to Quintero Stage IV. In consultation with a multi-disciplinary international team, fetoscopic laser photocoagulation was performed. The pregnancy progressed to delivery of female infants at 33 weeks gestation, who have achieved all developmental milestones at 2 years of age.

**Conclusions:**

We describe the multi-disciplinary effort to optimise the maternal condition to allow fetoscopic laser photocoagulation and continued management of the maternal and fetal conditions to a successful pregnancy outcome.

## Background

Monochorionic twin pregnancies are associated with an increased risk of fetal morbidity and mortality due to complications related to their chorionicity [1]. One of the most common complications of monochorionic pregnancy, and a reason for increased ultrasonographic surveillance, is Twin-Twin Transfusion Syndrome (TTTS), occurring in 10–20% of monozygous gestations [2]. Classification systems to categorise this condition, such as the Quintero Classification aid in the diagnosis and formulation of management plans [3].

As our population becomes more medically diverse, women are entering pregnancy with medical conditions which necessitate Obstetric Medicine expertise. Some complications, such as cardiac and haematological conditions can pose unique challenges to clinicians in both obstetrics, medicine and surgery. We describe the case of a woman diagnosed with stage IV TTTS in the setting of recurrent venous thromboembolic (VTE) disease.

## Case presentation

A 34 year old woman attended the perinatal medicine clinic of a tertiary maternity hospital in her second pregnancy at 8 weeks gestation owing to a history of recurrent VTE disease, where a dating ultrasound diagnosed a monochorionic twin pregnancy. She was a non-smoker, with no family history of VTE disease and a Body Mass Index of less than 35 kg/m^2^.

Her medical history was significant for multiple diagnoses of VTE disease over the preceding 10 years, despite negative thrombophilia testing. Bilateral pulmonary emboli (PE) were diagnosed following dental extraction (in conjunction with oral contraceptive pill use), followed 7 years later by a left above-knee deep vein thrombosis (DVT) and bilateral PE (in conjunction with a leg fracture and long-haul flight). A third episode of DVT and PE was identified at the time of a pregnancy loss. All episodes were treated with therapeutic anticoagulation. Subsequently, a temporary inferior vena cava (IVC) filter was inserted prior to an elective laparoscopic cholecystectomy, yet on removal of this, a thrombus was identified requiring further anticoagulation prior to removal.

One month following the IVC removal this lady had a positive pregnancy test and prophylactic LMWH was commenced followed by therapeutic LMWH on confirmation of a twin intrauterine pregnancy 2 weeks later. This dose was adjusted according to maternal weight and its efficacy was monitored by measuring trough and peak anti-factor Xa levels. At 16 weeks of gestation, anticoagulation was changed to twice daily enoxaparin due to low levels of anti-factor Xa, which indicated a rapid clearance of heparin.

As per guidance for monochorionic pregnancies [4], at 16 weeks gestation, fortnightly ultrasound surveillance was commenced. At 18 weeks gestation, Quintero stage I TTTS was diagnosed; Twin 1 was identified as the recipient twin with polyhydramnios and a large bladder while the donor twin (Twin 2) had oligohydramnios and a small bladder. Umbilical artery Doppler measurements for both twins were within normal limits at this time.

At this juncture, this lady was admitted to hospital for multi-disciplinary team involvement. Given the early gestation at which TTTS had developed and the potential for intervention, a permanent IVC filter was placed. The decision to place an IVC filter was made by the multi-disciplinary team due to the anticipated required interruption or reduction in anticoagulation which would increase the maternal risk of VTE disease at the time of fetoscopic surgery. Within 4 days, scan findings quickly progressed to Quintero stage IV TTTS. The recipient twin developed grossly abnormal cardiac function with evidence of fetal hydrops. The donor twin had anhydramnios, with an absent bladder and absent end diastolic flow of the umbilical artery Doppler.

Opinion was sought from national and international referral centres for consideration of laser photocoagulation. Fetal and maternal procedure-related risks were carefully considered and the potential risk of with-holding fetal treatment in the maternal interest was also discussed. The initial recommendations by a national centre for fetoscopic surgery was that due to the maternal medical co-morbidities and abnormal fetal cardiac function, with associated poor prognosis, laser photocoagulation should not be performed. Following re-assessment at the index hospital, a referral was made to a fetal medicine unit in a tertiary hospital in the United Kingdom (UK). Multidisciplinary discussion took place at this point with input from maternal medicine and haematology, as well as detailed patient and family counselling, discussed the risks of interrupting therapeutic LMWH versus the possible benefits to the fetuses of successful fetoscopic laser photocoagulation. Following this, it was decided to perform surgery following the interruption of therapeutic LMWH 24 h before surgery and recommence this at 4 h post-procedure.

Following her return from the UK, subsequent fetal surveillance scans were performed twice-weekly (assessing amniotic fluid status and umbilical artery Doppler measurements) which returned to normal pattern following treatment. In view of the previous complications, antenatal corticosteroids were administered at 24 weeks gestation and close maternal and fetal surveillance continued for the remainder of the pregnancy.

A detailed anatomical survey revealed persistent cardiomegaly and evidence of impaired cardiac contractility in the ex-recipient twin, however both fetal cardiac echocardiograms were reassuringly structurally normal. Fetal brain Magnetic Resonance Imaging (MRI) at 25 weeks gestation revealed no neurological abnormalities in either fetus.

Subsequently, pregnancy-induced hypertension was diagnosed at 32 weeks gestation. At this point growth for Twin 1 (ex-recipient) was 1.32 kg (less than the 2nd centile), with Twin 2 (ex-donor) noted to be 1.61 kg (11th centile). Bladder size and umbilical artery Doppler studies were normal at this juncture. Serial surveillance continued, and 7 days later, Twin 1 had absent end diastolic flow with abnormal ductus venosus flow. The decision was made to proceed with caesarean delivery in the fetal interests, and a multi-disciplinary team discussion including maternal-fetal medicine, obstetrics, haematology, anaesthesiology and neonatology was conducted to plan delivery. Later that day, 12 h following the therapeutic dose of enoxaparin, and under spinal anaesthesia, two live female infants were delivered, weighing 1.5 kg and 1.9 kg respectively. They were transferred to the Special Care Baby Unit in view of their antenatal course, prematurity and low birth weights. Half-dose anticoagulation was administered 6 h following the caesarean section, and full therapeutic dosage was re-instituted 24 h post-operatively.

The post-partum period was otherwise uncomplicated. She was reviewed by the haematology team at 6 weeks postpartum and converted to apixaban twice daily. She will now remain on life-long anticoagulation due to significant risks of recurrent VTE disease. Both twins were discharged on Day 30 of life, with normal cranial ultrasounds and were achieving appropriate developmental milestones at 6 weeks corrected age. Further follow-up was performed until 2 years of age, when both girls were maintaining their developmental milestones and were discharged from paediatric care.

## Discussion and conclusions

Our case describes a complex clinical situation of rapidly progressive TTTS with an associated poor prognosis in the absence of timely treatment, in a woman with a significant medical co-morbidity. While this lady had a high probability of suffering a further thromboembolic event in isolation of fetal complications, treatment in order to maximise fetal survival and minimise morbidity posed further clinical dilemmas. Any surgical procedure posed an increased haemorrhage risk, and concurrently propagated the incidence of thromboembolic events, both of which had maternal and fetal morbidity and mortality as a consequence. Therefore, careful multidisciplinary management was crucial to prevent deleterious consequences.

Despite being first described in 1687, the natural history and aetiology of TTTS is largely unknown. It is associated with a fetal mortality rate of 80–90% if untreated [5, 6] and neurological handicap affecting 10–30% of co-twin survivors following single twin intrauterine death [7]. Many different treatment strategies have been proposed and practiced, but fetoscopic laser photocoagulation is currently the primary treatment modality pursued [8].

Numerous studies have compared treatment strategies for TTTS, assessing success as well as risk. A comparison of amnioreduction and laser ablation involving over 142 cases demonstrated that those undergoing laser ablation were more likely to be free of major neurological complications at 6 months of age, with improved survival of Quintero stage III and IV (66% versus 44%) compared to the amnioreduction group [9].

Despite a relatively small body of evidence, fetoscopic laser photocoagulation is considered by most experts to be the best approach for stage II - IV TTTS in pregnancies less than 26 weeks gestation [1, 8, 10]. In Ireland, fetoscopic laser photocoagulation was reviewed in 2015, with 105 cases performed over a 5 year period [11]. Dual survival rates overall were 47%, and perinatal survival of at least one infant was 75%; however only 5 (5%) cases were classified and treated as stage IV. When compared to international data, where stage IV procedures account for a higher number of cases performed (27 (16.5%)} [12], it was felt that a referral centre with more experience provided increased survival and reduced neurological morbidity in this scenario.

Examining the maternal factors in this case, VTE disease is known to affect women in pregnancy and the puerperium approximately 10 times more frequently when compared to an age-matched non-pregnant cohort of women [13]. The treatment of recurrent VTE disease in pregnancy is complex and debated, with some authors recommending retrievable filters over permanent filters given the age profile of the pregnant woman coupled with limited available follow-up data for this cohort [14]. Given the paucity of research in this area, treatment is still largely experimental and needs further investigation, potentially in the setting of a randomised controlled trial; however both the feasibility and ethical acceptability of this would make this difficult. In fields other than obstetrics, the placement of IVC filters does remain an unsubstantiated indication for the treatment of recurrent VTE disease. In cases of recurrent VTE and prolonged anti-coagulation, vitamin D and calcium supplementation may need to be considered, but further high grade evidence would be required in order to make this a formal recommendation [15, 16].

We describe a complex case with maternal and fetal complications and considerations that evolved throughout the pregnancy. Our case is unique, in that coupled with the rapidly progressive early development of TTTS to Quintero stage IV, this patient had a very high risk for recurrent VTE entering pregnancy, and was successfully managed for both indications. In addition, multiple pregnancy, polyhydramnios, antenatal and puerperal surgical procedures combined with prolonged hospitalisation increased our patients’ risk of recurrent VTE disease significantly. Multidisciplinary and multi-centre management was crucial to optimising maternal and fetal outcomes. Haematological guidance on dosage of anticoagulation and amendments to the regime at the time of fetoscopic surgery and caesarean section were crucial, and given the gravity of both situations required timely and clear decision making and communication.

Through expert assessment of clinical complications both in isolation and in tandem, we demonstrate how complex high-risk and challenging medical scenarios can be successfully managed, with assistance from the local, national and international expertise in time-critical scenarios.

## Data Availability

Not applicable

## Abbreviations

DVT: Deep vein thrombosis
IVC: Inferior vena cava
LMWH: Low Molecular Weight Heparin
PE: Pulmonary Embolus
TTTS: Twin Twin Transfusion Syndrome
VTE: Venous thromboembolic
